# Levothyroxine Monotherapy Cannot Guarantee Euthyroidism in All Athyreotic Patients

**DOI:** 10.1371/journal.pone.0022552

**Published:** 2011-08-01

**Authors:** Damiano Gullo, Adele Latina, Francesco Frasca, Rosario Le Moli, Gabriella Pellegriti, Riccardo Vigneri

**Affiliations:** Endocrine Unit, Department of Clinical and Molecular Biomedicine, University of Catania Medical School, Garibaldi-Nesima Hospital, Catania, Italy; Cardiff University, United Kingdom

## Abstract

**Context:**

Levothyroxine monotherapy is the treatment of choice for hypothyroid patients because peripheral T4 to T3 conversion is believed to account for the overall tissue requirement for thyroid hormones. However, there are indirect evidences that this may not be the case in all patients.

**Objective:**

To evaluate in a large series of athyreotic patients whether levothyroxine monotherapy can normalize serum thyroid hormones and thyroid-pituitary feedback.

**Design:**

Retrospective study.

**Setting:**

Academic hospital.

**Patients:**

1,811 athyreotic patients with normal TSH levels under levothyroxine monotherapy and 3,875 euthyroid controls.

**Measurements:**

TSH, FT4 and FT3 concentrations by immunoassays.

**Results:**

FT4 levels were significantly higher and FT3 levels were significantly lower (p<0.001 in both cases) in levothyroxine-treated athyreotic patients than in matched euthyroid controls. Among the levothyroxine-treated patients 15.2% had lower serum FT3 and 7.2% had higher serum FT4 compared to euthyroid controls. A wide range of FT3/FT4 ratios indicated a major heterogeneity in the peripheral T3 production capacity in different individuals. The correlation between thyroid hormones and serum TSH levels indicated an abnormal feedback mechanism in levothyroxine-treated patients.

**Conclusions:**

Athyreotic patients have a highly heterogeneous T3 production capacity from orally administered levothyroxine. More than 20% of these patients, despite normal TSH levels, do not maintain FT3 or FT4 values in the reference range, reflecting the inadequacy of peripheral deiodination to compensate for the absent T3 secretion. The long-term effects of chronic tissue exposure to abnormal T3/T4 ratio are unknown but a sensitive marker of target organ response to thyroid hormones (serum TSH) suggests that this condition causes an abnormal pituitary response. A more physiological treatment than levothyroxine monotherapy may be required in some hypothyroid patients.

## Introduction

The elective treatment for hypothyroid patients requiring replacement therapy is oral administration of synthetic levothyroxine. Although the normal thyroid gland secretes both T4 and T3, the peripheral conversion of ingested levothyroxine into T3 is believed to provide the overall required amount of thyroid hormones necessary to reach the euthyroid state, as indicated by serum TSH levels within the normal range.

Over the years, it has been recognized that some hypothyroid patients do not obtain a condition of well-being under levothyroxine monotherapy and prefer T3–T4 combined treatment [1], [2]. Moreover, to achieve normal serum TSH levels, the administered levothyroxine dose must be sufficient enough to raise serum free T4 (FT4) concentration to the upper normal range [3], [4].

These observations suggest that some levothyroxine-treated hypothyroid patients may have an insufficient T3 production and a reduced T3/T4 ratio. Their peripheral tissues may be exposed to an imbalanced availability of circulating thyroid hormones. The consequences of this condition are not clear. Tissues take up iodothyronines by surface transporters and deiodinate the pre-hormone T4 to the active hormone T3. Different tissues, however, express different machinery (in terms of both quality and quantity of transporters and deiodinases) for producing intracellular T3 and differ in their ability to compensate for an abnormal T3/T4 availability [5], [6]. Because of tissue heterogeneity, pituitary secreted TSH may not reflect what happens in other target tissues and, therefore, in some instances serum TSH may not be an appropriate indicator of peripheral tissue euthyroidism [7], [8], [9]. This has been demonstrated in thyroidectomized rats that, when infused with levothyroxine alone, do not reach euthyroidism at all tissue level [5]. Although similar studies have not been carried out in humans, it is possible that the unsatisfactory health condition of some hypothyroid patients, even when well compensated in terms of TSH serum levels [10], [11], might be due to insufficient T3 availability in one or more peripheral tissues.

Athyreotic patients do not secrete endogenous thyroid hormones and all circulating T4 and T3 originate from replacement treatment with levothyroxine. These patients, therefore, are an ideal model to study peripheral tissues' capacity to produce the biologically active hormone T3 from the exogenous prehormone T4.

We evaluated FT4 and free T3 (FT3) serum levels in a large series of athyreotic patients under oral levothyroxine monotherapy. All patients were euthyroid on the basis of their TSH serum level within the normal range. Compared to euthyroid controls, one fifth of the athyreotic patients treated with levothyroxine monotherapy had abnormal values of either FT3 or FT4. Moreover, an abnormal thyroid-pituitary feedback was observed in these patients, with a reduced sensitivity compared to euthyroid controls.

## Methods

### Subjects

Serum TSH, FT4 and FT3 values were retrospectively analyzed in a continuous series of 3,473 athyreotic patients who were all followed at our Thyroid Clinic (University of Catania, Garibaldi-Nesima Hospital, Catania, Italy) from 2000–2007, after undergoing total thyroidectomy for thyroid cancer. All were treated with levothyroxine monotherapy at a stable dosage since at least 3 months prior to our examination.

Of these patients, those under “suppressive” levothyroxine treatment (n = 1,662, TSH<0.4 mU/L) because of their thyroid cancer risk category, were excluded. Patients with normal (0.4–4.0 mU/L) serum TSH levels under levothyroxine monotherapy were included (n = 1,881) and, out of them, 598 (33%) had also been treated with ^131^iodine to ablate residual thyroid tissue. Patients were subdivided according to gender (F = 1,530 and M = 281) and age (1,298<60 y and 513≥60 y). All patients were disease-free from thyroid cancer as judged by serum thyroglobulin levels (≤0.5 ng/ml after levothyroxine treatment withdrawal) and had a negative ultrasound neck examination and a negative total body scan, if required. All were treated with a levothyroxine dosage adequate to maintain TSH serum concentrations within the normal range (0.4–4.0 mU/L). Levothyroxine was always recommended to be ingested in the morning, with water and in a fasting state, and hormones were measured from a serum sample obtained in the morning, before levothyroxine ingestion. When more than one set of measurements was available for the same patient, the most recent sample was chosen.

Serum TSH, FT4 and FT3 values were retrospectively analyzed also in a continuous series of 3,875 euthyroid subjects, all resident in areas of sufficient iodine intake [12]. These subjects were referred to our Thyroid Clinic in the same time period because of a non-functioning benign thyroid nodule <20 mm diameter and they showed no clinical or laboratory sign of altered thyroid function. Clinically euthyroid subjects with serum TSH<0.4 or >4.0 mU/L were excluded under suspicion of subclinical hyper- or hypo-thyroidism. Subjects positive for anti-TPO and/or anti-Tg antibodies and/or with hyperechogenicity or pseudo-nodular pattern at thyroid ultrasound examination were also excluded. As for athyreotic patients, also subjects in this group were subdivided by gender (F = 3,224, M = 651) and age (2,927<60 y and 948≥60 y). None of these control subjects had ever been treated with thyroid hormones or antithyroid drugs.

In both groups, patients with clinically significant organ diseases (renal failure and severe liver disease) or recent (less than 6 months) major surgery or illness or with familial thyroid disease or being treated with drugs known to interfere with thyroid function or thyroid hormone metabolism were excluded. In particular, patients using amiodarone, propranolol, steroids or with recent (less than one year) use of iodinated contrast material were excluded to avoid potential interferences with deiodinase activity. The study was approved by the local Ethics Committee.

### Hormone measurements

All hormone measurements were determined in our Hospital's central laboratory. Serum FT3, FT4 and TSH levels were measured by automated microparticle enzyme immunoassays (Abbott AxSYM-MEIA, Abbott Park, IL, USA) with inter-assay coefficients of variation of less than 10% over the analytical ranges of 1.7–46.0 pmol/L for FT3, 5.15–77.0 pmol/L for FT4 and 0.03–10.0 mU/L, for TSH. The within-run and between–run precisions for the FT3, FT4 and TSH assays showed coefficients of variation <5%.

### Statistical Analysis

The TSH serum value distribution was non-Gaussian, with a skewness toward higher values. Also, for FT4 and FT3 serum values the normal distribution assumptions were not satisfied using the D'Agostino-Pearson test. Therefore, non-parametric tests were used and results were reported as a median value and an interquartile range (IQR, 25^th^–75^th^ quartiles). The Mann-Whitney non-parametric test for unpaired values was used to determine statistical significance. To calculate linear regression coefficients, TSH data were log transformed to normalize the skewed distribution. Data were analyzed with the Prism software package (GraphPad, USA). Differences between groups, after adjusting for several covariates, were evaluated by the covariance analysis (ANCOVA) test using SPSS (version 15.0; SPSS, Chicago, IL, USA).

## Results

### TSH, FT4 and FT3 serum values in euthyroid controls

**Figure 1:**
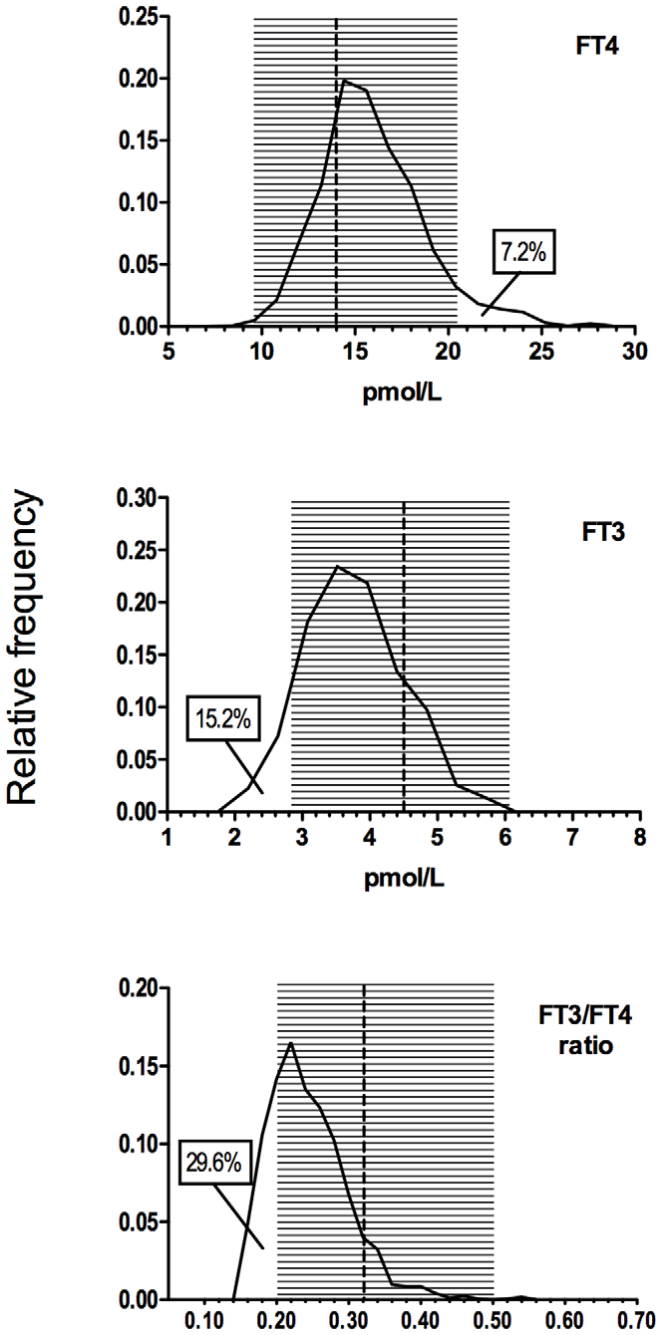
Free thyroid hormones and FT3/FT4 ratio frequency distribution. FT3 and FT4 serum levels and FT3/FT4 ratio distribution in 1,811 athyreotic patients under levothyroxine (L-T4) monotherapy. Shaded areas indicate the normal range (2.5–97.5 percentiles) calculated in 3,875 euthyroid controls. Vertical dotted lines indicate the median of the normal values. Percentages indicate the patients with values under or above the normal values.

In our series of 3,875 euthyroid adults, median serum TSH value was 1.40 mU/L (interquartile range, IQR = 0.90–2.10); median FT4 value was 13.8 pmoles/L (IQR = 12.0–15.4); and median FT3 value was 4.47 pmoles/L (IQR = 3.85–4.94) (Table 1). In this euthyroid population the normal range (2.5–97.5 percentiles) was 9.0–20.6 pmoles/L for FT4 and 2.9–6.0 pmoles/L for FT3. The median individual FT3/FT4 ratios was 0.32 (IQR = 0.27–0.37) (Figure 1).

**Table 1:** TSH, FT4, FT3 levels in studied subjects by age and gender.

		F/M	no.	Age (yrs)	TSH (mU/L)	FT4 (pmol/L)	FT3 (pmol/L)	FT3/FT4 ratio	L-T4 (µg/Kg/d)
**Euthyroid controls**		4.9∶1	3875	49 (37–61)	1.40 (0.90–2.10)	13.8 (12.0–15.4)	4.47 (3.85–4.94)	0.32 (0.27–0.37)	
Females	all		3224	49 (37–61)	1.40 (0.90–2.10)	13.6 (11.8–15.4)	4.40 (3.85–4.93)	0.32 (0.27–0.37)	
	≤60 yr		2457	45 (36–53)	1.41 (0.90–2.17)	13.4 (11.6–15.4)†	4.44 (3.85–4.93)	0.32 (0.27–0.37)	
	>60 yr		767	68 (64–72)	1.29 (0.80–1.96)	14.2 (12.2–15.4)	4.31 (3.85–4.91)	0.30 (0.26–0.36)§	
Males	all		651	51 (35–64)	1.29 (0.80–1.94)	14.2 (12.5–15.9)	4.62 (4.16–5.30)	0.33 (0.28–0.39)‡	
	≤60 yr		470	45 (37–52)	1.30 (0.85–2.00)*	14.2 (12.5–16.0)	4.79 (4.24–5.40)*	0.33 (0.28–0.39)	
	>60 yr		181	68 (64–73)	1.20 (0.75–1.80)*	14.2 (12.4–15.6)	4.47 (4.00–5.08)*	0.31 (0.26–0.36)§	
**L-T4 treated pts**		5.4∶1	1811	51 (42–61)	1.20 (0.69–2.20)∥	15.4 (14.2–17.6)∥	3.70 (3.23–4.31)∥	0.24 (0.20–0.28)∥	1.59 (1.36–1.86)
Females	all		1530	52 (42–61)	1.20 (0.69–2.20)	15.4 (14.2–17.5)	3.70 (3.23–4.29)	0.24 (0.20–0.27)	1.59 (1.34–1.84)
	≤60 yr		1088	46 (39–53)	1.12 (0.60–2.10)	15.4 (14.2–17.0)	3.70 (3.23–4.31)	0.24 (0.21–0.28)	1.64 (1.40–1.93)
	>60 yr		442	66 (63–72)	1.20 (0.70–2.31)§	15.4 (14.2–18.0)	3.70 (3.16–4.16)	0.23 (0.20–0.26)§	1.43 (1.23–1.67)
Males	all		281	48 (35–64)	1.20 (0.69–2.40)	15.4 (14.2–18.0)	3.87 (3.47–4.40)	0.25 (0.21–0.28)‡	1.63 (1.41–1.92)
	≤60 yr		210	45 (36–52)	1.10 (0.64–2.40)	15.4 (14.2–18.0)	4.00 (3.54–4.47)¶	0.25 (0.21–0.29)	1.73 (1.47–1.98)
	>60 yr		71	67 (63–73)	1.40 (0.80–2.60)	15.5 (14.2–17.0)	3.70 (3.39–4.14)§	0.24 (0.20–0.27)	1.43 (1.37–1.67)

Values indicate median and interquartile ranges (IQR). FT3 = free triiodothyronine; FT4 = free thyroxine; TSH = thyroid-stimulating hormone; L-T4 = levothyroxine.

*p<0.001: males *vs.* females in the same age groups.

† p<0.001: females≤60 yr *vs*. females>60 yr and males, both age groups.

‡ p = 0.027: males *vs.* females.

§ p<0.001: ≤60 yr *vs.* >60 yr of the same gender.

∥ p<0.001: L-T4 treated athyreotic patients *vs.* euthyroid controls.

¶ p<0.001: males ≤60 yr vs. females in the same age range.

**Figure 2:**
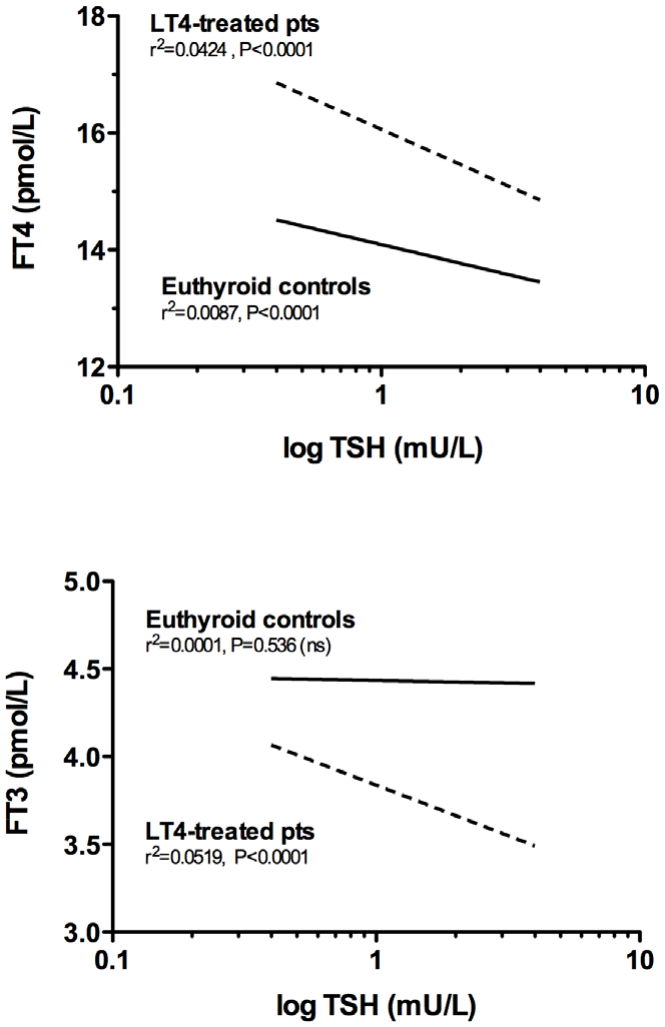
Correlation between TSH and free thyroid hormones in euthyroid controls and in athyreotic patients. The correlation between TSH serum levels (log values) and FT3 and FT4 serum levels in 3,875 euthyroid controls (solid lines) and 1,811 athyreotic patients under levothyroxine monotherapy (dotted lines). The linear regression equations between FT4 and log TSH levels in the euthyroid controls and the levothyroxine (L-T4)-treated patients were *y* = 14.0−1.1*x* (95%: slope −1.4 to −0.74) and *y* = 16.1−2.01*x* (95% CI: slope −2.48 to −1.53), respectively. The same curve fitting analysis was used between FT3 and log-TSH levels and resulted in the following: *y* = 4.4−0.029*x* (95% CI: slope −0.128 to 0.063) for euthyroid controls and *y* = 3.84−0.575*x* (95% CI: slope −0.697 to −0.453) for L-T4-treated patients. R square and *P* values are reported in the graph.

Hormone values were influenced by both gender and age. FT3 values were lower and TSH values were higher in females compared to males of the corresponding age range (Table 1). Also FT4 values in women younger than 60 yr were lower than those observed in men and, in this group of women, median FT4 value was significantly lower than that of older women and of men in both age groups (*P*<0.001 in both cases) (Table 1). These observations are in agreement with previous data on the effects of age and gender differences on serum free thyroid hormone values [13], [14]. Therefore, in the euthyroid controls the FT3/FT4 ratio was higher in males relative to females and in individuals younger than 60 relative to older subjects of the same gender (Table 1). Using a linear regression analysis model, in the euthyroid controls TSH serum values were inversely correlated with the serum FT4 values (r^2^ = 0.0087, slope −1.1, *P*<0.0001) but not with the FT3 serum values (r^2^ = 0.0001, slope −0.0029, *P* = 0.536) (Figure 2).

### TSH, FT4 and FT3 serum values in athyreotic patients under levothyroxine monotherapy

In the 1,811 athyreotic patients with normal serum TSH values (0.4–4.0 mU/L) under levothyroxine monotherapy, the median serum TSH was 1.20 mU/L (IQR = 0.69–2.20), the median serum FT4 value was 15.4 pmoles/L (IQR = 14.2–17.6, *P*<0.001 compared to euthyroid controls) and the median serum FT3 value was 3.70 pmoles/L (IQR = 3.73–4.31, *P*<0.001 relative to controls). The median individual FT3/FT4 ratio was 0.24 (0.20–0.28, *P*<0.001) (Table 1). These significant differences were observed also when patients were subdivided according to age and gender and then compared to age and gender matched controls.

In levothyroxine–treated athyreotic patients, the effect of age and gender on hormone levels was different from euthyroid controls. As expected, in athyreotic patients the FT4 serum levels, which are dependent on the ingested levothyroxine, were similar in all age and gender groups. In contrast, the FT3 values, which depend on peripheral deiodination of the ingested levothyroxine, were significantly higher in males younger than 60 yrs compared to older males and to females, as observed in the euthyroid controls (Table 1).

**Figure 3:**
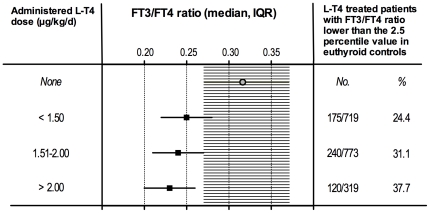
FT3/FT4 ratio in levothyroxine-treated athyreotic patients at different daily dose. Median FT3/FT4 ratio in levothyroxine (L-T4) treated athyreotic patients with respect to the administered daily dose of L-T4. The median and interquartile range in the euthyroid controls are indicated by the shaded area. The number of patients with a FT3/FT4 ratio lower than the 2.5 percentile of the euthyroid controls is indicated in the right panel.

Among the athyreotic patients, despite normal serum TSH levels, 15.2% had serum FT3 levels lower than the normal range and 7.2% had FT4 levels higher than the normal range (Figure 1). The percentage of athyreotic patients with FT3 serum levels lower than the normal range was 8.6% in males and 16.4% in females (*P* = 0.044). The percentage of FT3 serum values lower than the normal range was also lower in patients younger than 60 yrs (14.3%) compared to the older patients (17.4%) but this difference was not statistically significant. Finally, 29.6% of the levothyroxine-treated athyreotic patients had FT3/FT4 ratios lower than the normal range (<2.5 percentile in the euthyroid controls) (Figure 1). The percentage of patients with an abnormally low FT3/FT4 ratio progressively increased with increasing levothyroxine dose, reaching 37.7% in the 319 patients treated with >2.0 µg/kg/d of levothyroxine (Figure 3).

### Thyroid-pituitary feedback in athyreotic patients

When the 1,811 levothyroxine-treated athyreotic patients with normal TSH serum value were subdivided in groups according to their TSH level and then matched to euthyroid subjects with comparable TSH levels, the median FT4 serum level was significantly higher and the median FT3 serum level was significantly lower in each levothyroxine-treated group compared to TSH-matched euthyroid controls (Table 2). These differences remained significant after the data were adjusted for age, gender and TSH values (*P*<0.001, ANCOVA test). At a similar median serum FT4 level the median serum TSH value was much lower in the euthyroid controls than in the levothyroxine–treated patients (Table 2). Moreover, the linear regression analysis (Figure 2) between TSH and FT4 serum values was much steeper in levothyroxine-treated patients who, therefore, required a significantly greater FT4 serum change to obtain the same feed-back effect on pituitary TSH-secreting cells. This difference may be explained by the different FT3 levels in the two conditions, with values significantly lower in the levothyroxine-treated athyreotic patients (Table 2). When serum FT4 levels decreased, euthyroid controls partially compensated by maintaining a nearly constant FT3 serum level (Figure 2), a likely consequence of increased T3 de novo synthesis in the thyroid. In contrast, in levothyroxine-treated patients the FT3 levels changed in parallel with the FT4 level decrease but the magnitude of serum TSH change was smaller suggesting a less sensitive response of thyrotropic cells to thyroid hormones.

**Table 2:** TSH, FT4, FT3 levels in euthyroid controls and in levothyroxine-treated athyreotic patients subdivided according to TSH levels.

TSH levels (mU/L)					
	Euthyroid controls (n = 3875)				
	n.	TSH (mU/L)	FT4 (pmol/L)	FT3 (pmol/L)	FT3/FT4 ratio
**0.40–1.00**	1306	0.70 (0.57–0.90)	14.2 (12.5–15.7)	4.47 (3.85–5.00)	0.31 (0.27–0.36)
**1.01–1.50**	878	1.30 (1.16–1.40)	13.9 (12.1–15.4)	4.34 (3.85–4.93)	0.31 (0.27–0.37)
**1.51–2.00**	625	1.79 (1.62–1.90)	13.8 (12.0–15.4)	4.47 (3.85–4.93)	0.31 (0.27–0.37)
**2.01–2.50**	402	2.26 (2.11–2.40)	13.4 (11.6–15.4)	4.47 (3.85–4.94)	0.32 (0.27–0.37)
**2.51–4.00**	664	3.10 (2.70–3.70)	12.9 (11.6–15.0)	4.47 (3.85–5.00)	0.33 (0.28–0.39)

	Athyreotic patients (n = 1811)				
	n.	TSH (mU/L)	FT4 (pmol/L)	FT3 (pmol/L)	FT3/FT4 ratio
**0.40–1.00**	793	0.60 (0.47–0.80)	15.7 (14.2–18.0)*	3.96 (3.39–4.47)*	0.24 (0.21–0.27)*
**1.01–1.50**	326	1.20 (1.12–1.40)	16.2 (14.2–18.0)*	3.85 (3.39–4.37)*	0.24 (0.20–0.27)*
**1.51–2.00**	183	1.80 (1.67–1.90)	15.4 (14.2–16.7)*	3.65 (3.13–4.15)*	0.23 (0.20–0.28)*
**2.01–2.50**	135	2.30 (2.16–2.38)	14.9 (13.3–17.6)*	3.60 (3.13–4.04)*	0.24 (0.20–0.28)*
**2.51–4.00**	374	3.32 (2.90–4.07)	14.2 (12.9–15.4)*	3.39 (2.96–3.90)*	0.23 (0.20–0.28)*

Values indicate median and interquartile ranges (IQR). FT3 = free triiodothyronine; FT4 = free thyroxine; TSH = thyroid-stimulating hormone; L-T4 = levothyroxine.

*p<0.001: L-T4-treated athyreotic patients vs. euthyroid controls with similar serum TSH levels.

## Discussion

In a very large series of athyreotic patients we observe that, to obtain euthyroidism as indicated by TSH normalization, FT4 values must be significantly higher than in euthyroid controls. Conversely, in spite of the increased FT4 levels, FT3 serum values are significantly lower than in normal subjects, suggesting that peripheral T4 to T3 conversion may not be sufficient to maintain a normal FT3/FT4 ratio under levothyroxine monotherapy. The decreased FT3/FT4 ratio is more marked in a subset of patients (approximately one third in our study) that do not reach a serum FT3/FT4 ratio within the reference range observed in euthyroid controls (Figure 1). These patients, therefore, live in a chronic condition of abnormal thyroid hormone availability for the peripheral tissues, even if the administered levothyroxine dose is able to maintain the serum TSH within the normal range. This condition is more frequent in female and aged patients, indicating a significant gender and age influence on the individual capacity to produce T3 from exogenous levothyroxine.

These abnormalities have already been reported [3], [4], [15] but their relevance has been minimized in the absence of clear evidence that they may have clinical consequences. Therefore, based on the evidence that (i) all hypothyroid patients can reach normal TSH serum levels adjusting the levothyroxine dose; (ii) that most (although not all) patients can maintain a condition of general well-being under levothyroxine monotherapy and (iii) that in many studies a short period of T4-T3 combination therapy has not caused a significant improvement in a variety of symptoms, the conclusion has been drown that levothyroxine monotherapy must be the standard treatment for all hypothyroid patients [16]. It should be underlined, however, that no long-term study is available to assess that the abnormal circulating thyroid hormone ratio has no adverse effect and that even short-term studies indicate that some hypothyroid patients do not reach a condition of well-being and emotional satisfaction under levothyroxine monotherapy [17]. Their condition may ameliorate under a combined T4-T3 treatment [1], [18], [19], [20].

Studying a sufficiently large series of thyroidectomized patients without residual thyroid tissue we now observe that, despite normal serum TSH, under levothyroxine monotherapy approximately one fifth of them has either FT3 or FT4 serum levels outside the reference range in euthyroid controls. Moreover, also among levothyroxine treated patients with normal circulating hormones, normality can be questioned in same cases. In the study by Jonklaas et al [21], aimed at demonstrating that normal T3 serum values can be obtained in all athyreotic patients under levothyroxine replacement therapy, the normal T3 value, similar to the pre-thyroidectomy value, is an average value that includes values changed more than 30% in respect to the previous T3 serum levels and is obtained at the expenses of an average 50% increase of TSH and 30% increase of FT4. Patients that, in spite of having hormone values in the normal range show such marked changes in their TSH and thyroid hormones pre-surgery values can be considered truly euthyroid [22]? Individuals with changes of similar magnitude in biologic parameters like glycemia or hearth rate would not be considered normal because, although no clinical signs or symptoms are present, deleterious consequences will appear in the long run.

The major source of T3 within peripheral tissues comes from the circulating T3 pool and the variable portion coming from locally deiodinated T4 within each tissue might be not adequate according to animal experiments [5]. The long-term effect of this abnormal condition are unknown and the proof of concept that no benefit can derive from combined T4/T3 treatment cannot be affirmed on the basis of short-term studies.

Analyzing the correlation between TSH and thyroid hormones serum levels in a large series of athyreotic patients under levothyroxine monotherapy, we observe that in these patients the pituitary response is significantly different from normal, being the pituitary feedback much less sensitive than in euthyroid controls (Figure 2). This evidence indicates that the effect of thyroid hormones at pituitary level is altered in athyreotic patients under levothyroxine monotherapy. A practical consequence of this finding is that normalization of serum TSH cannot be always considered an appropriate marker of euthyroidism in levothyroxine-treated patients.

Athyreotic individuals show a relevant heterogeneity in their capacity to deiodinate exogenous levothyroxine to T3 as indicated by the variability of FT3 serum concentrations in patients with similar FT4 levels. While most patients under levothyroxine monotherapy will reach normal TSH serum levels, a normal FT3/FT4 ratio and also full improvement of signs and symptoms, a subset of patients is unable to convert the ingested levothyroxine into an adequate amount of T3. This may happen for a variety of reasons, including congenital or acquired deficiency of deiodinase function [23], [24], [25] and also abnormal thyroid hormone metabolism independent from deiodination [26]. Whether these patients are the ones that do not reach well-being under levothyroxine monotherapy and could benefit from a combined, more physiological T4/T3 treatment cannot be inferred from our study that has not measured the well-being state or the level of satisfaction in these patients. Our study has also the limitations inherent to retrospective observational studies, in which the interference of unrecognized confounders cannot be excluded in the comparison between studied and control groups. Finally, the lack of measurements of tissue deiodinases and of thyroid hormone metabolites (like reverse triiodothyronine) leave to speculation the mechanisms and the metabolic pathways involved in the variability of the FT3/FT4 ratio in L-thyroxine treated patients.

he insufficient T3 peripheral production cannot be appropriately corrected by increasing levothyroxine dose because the inhibitory effect of elevated T4 on type II deiodinase [27], [28] will end up increasing the imbalance of the circulating FT3/FT4 ratio (Figure 3).

In conclusion, athyreotic patients treated with levothyroxine monotherapy show a highly heterogeneous capacity of T3 production and about one fifth of those, despite normal TSH levels, do not maintain FT3 or FT4 values within the reference range. A reduced thyrotropic cells sensitivity to thyroid hormones is also present in levothyroxine-treated athyreotic patients. These observations do not allow to take for granted the conclusions, drawn from short-term studies, that levothyroxine monotherapy is adequate for all hypothyroid patients [16], considering that even subtle abnormalities might have important consequences when lasting for many years or decades. The question whether a subgroup of hypothyroid patients requires a more physiological treatment is still not answered and requires further studies [29], [30], [31].

## References

[1] Bunevicius R, Kazanavicius G, Zalinkevicius R, Prange AJ (1999). Effects of thyroxine as compared with thyroxine plus triiodothyronine in patients with hypothyroidism.. N Engl J Med.

[2] Appelhof BC, Fliers E, Wekking EM, Schene AH, Huyser J (2005). Combined therapy with levothyroxine and liothyronine in two ratios, compared with levothyroxine monotherapy in primary hypothyroidism: a double-blind, randomized, controlled clinical trial.. J Clin Endocrinol Metab.

[3] Woeber KA (2002). Levothyroxine therapy and serum free thyroxine and free triiodothyronine concentrations.. J Endocrinol Invest.

[4] Iverson JF, Mariash CN (2008). Optimal free thyroxine levels for thyroid hormone replacement in hypothyroidism.. Endocr Pract.

[5] Escobar-Morreale HF, Obregón MJ, Escobar del Rey F, Morreale de Escobar G (1995). Replacement therapy for hypothyroidism with thyroxine alone does not ensure euthyroidism in all tissues, as studied in thyroidectomized rats.. J Clin Invest.

[6] Dayan CM, Panicker V (2009). Novel insights into thyroid hormones from the study of common genetic variation.. Nat Rev Endocrinol.

[7] Meier C, Trittibach P, Guglielmetti M, Staub JJ, Müller B (2003). Serum thyroid stimulating hormone in assessment of severity of tissue hypothyroidism in patients with overt primary thyroid failure: cross sectional survey.. BMJ.

[8] Zulewski H, Müller B, Exer P, Miserez AR, Staub JJ (1997). Estimation of tissue hypothyroidism by a new clinical score: evaluation of patients with various grades of hypothyroidism and controls.. J Clin Endocrinol Metab.

[9] Alevizaki M, Mantzou E, Cimponeriu AT, Alevizaki CC, Koutras DA (2005). TSH may not be a good marker for adequate thyroid hormone replacement therapy.. Wien Klin Wochenschr.

[10] Saravanan P, Chau WF, Roberts N, Vedhara K, Greenwood R (2002). Psycological well-being in patients on ‘adequate’ doses of l-thyroxine: results of a large, controlled community-based questionnaire study.. Clin Endocrinol (Oxf.).

[11] Samuels MH, Schuff KG, Carlson NE, Carello P, Janowsky JS (2007). Health status, psychological symptoms, mood, and cognition in L-thyroxine-treated hypothyroid subjects.. Thyroid.

[12] Regalbuto C, Scollo G, Pandini G, Ferrigno R, Pezzino V (2010). Effects of prophylaxis with iodised salt in an area of endemic goitre in north-eastern Sicily.. J Endocrinol Invest.

[13] Davey R (1997). Thyroxine, thyrotropin, and age in a euthyroid hospital patient population.. Clin Chem.

[14] Kratzsch J, Fiedler GM, Leichtle A, Brügel M, Buchbinder S (2005). New reference intervals for thyrotropin and thyroid hormones based on National Academy of Clinical Biochemistry criteria and regular ultrasonography of the thyroid.. Clin Chem.

[15] Fadeyev VV, Morgunova TB, Sytch JP, Melnichenko GA (2005). TSH and thyroid hormones concentrations in patients with hypothyroidism receiving replacement therapy with L-thyroxine alone or in combination with L-triiodothyronine.. Hormones (Athens).

[16] Grozinsky-Glasberg S, Fraser A, Nahshoni E, Weizman A, Leibovici L (2006). Thyroxine-triiodothyronine combination therapy versus thyroxine monotherapy for clinical hypothyroidism: meta-analysis of randomized controlled trials.. J Clin Endocrinol Metab.

[17] Wekking EM, Appelhof BC, Fliers E, Schene AH, Huyser J (2005). Cognitive functioning and well-being in euthyroid patients on thyroxine replacement therapy for primary hypothyroidism.. Eur J Endocrinol.

[18] Ma C, Xie J, Huang X, Wang G, Wang Y (2009). Thyroxine alone or thyroxine plus triiodothyronine replacement therapy for hypothyroidism.. Nucl Med Commun.

[19] Nygaard B, Jensen EW, Kvetny J, Jarløv A, Faber J (2009). Effect of combination therapy with thyroxine (T4) and 3,5,3′-triiodothyronine versus T4 monotherapy in patients with hypothyroidism, a double-blind, randomised cross-over study.. Eur J Endocrinol.

[20] Panicker V, Saravanan P, Vaidya B, Evans J, Hattersley AT (2009). Common variation in the DIO2 gene predicts baseline psychological well-being and response to combination thyroxine plus triiodothyronine therapy in hypothyroid patients.. J Clin Endocrinol Metab.

[21] Jonklaas J, Davidson B, Bhagat S, Soldin SJ (2008). Triiodothyronine levels in athyreotic individuals during levothyroxine therapy.. JAMA.

[22] Cooper DS (2008). Thyroxine monotherapy after thyroidectomy: coming full circle.. JAMA.

[23] Köhrle J, Jakob F, Contempré B, Dumont JE (2005). Selenium, the thyroid, and the endocrine system.. Endocr Rev.

[24] De Jong FJ, Peeters RP, Den Heijer T, Van der Deure WM, Hofman A (2007). The association of polymorphisms in the type 1 and 2 deiodinase genes with circulating thyroid hormone parameters and atrophy of the medial temporal lobe.. J Clin Endocrinol Metab.

[25] Panicker V, Cluett C, Shields B, Murray A, Parnell KS (2008). A common variation in deiodinase 1 gene DIO1 is associated with the relative levels of free thyroxine and triiodothyronine.. J Clin Endocrinol Metab.

[26] Visser TJ, van Buuren JC, Rutgers M, Eelkman Rooda SJ, de Herder WW (1990). The role of sulfation in thyroid hormone metabolism.. Trends Endocrinol Metab.

[27] Bianco AC, Kim BW (2006). Deiodinases: implications of the local control of thyroidhormone action.. J Clin Invest.

[28] Bianco AC, Salvatore D, Gereben B, Berry MJ, Larsen PR (2002). Biochemistry, cellular and molecular biology, and physiological roles of the iodothyronine selenodeiodinases.. Endocr Rev.

[29] Wiersinga WM (2001). Thyroid hormone replacement therapy.. Horm Res.

[30] Hennemann G, Docter R, Visser TJ, Postema PT, Krenning EP (2004). Thyroxine plus low-dose, slow-release triiodothyronine replacement in hypothyroidism: proof of principle.. Thyroid.

[31] Acosta BM, Bianco AC (2010). New insights into thyroid hormone replacement therapy.. F1000 Med Rep May.

